# Prevalence and Associated Factors with Poor Sleep Quality in Non-Professional Caregivers

**DOI:** 10.3390/jcm11030719

**Published:** 2022-01-29

**Authors:** Miguel A. Simón, Ana M. Bueno, Vanessa Blanco, Patricia Otero, Fernando L. Vázquez

**Affiliations:** 1 Health Psychology Research Unit, Department of Psychology, University of A Coruña, 15071 A Coruña, Spain; ana.bueno@udc.es (A.M.B.); patricia.otero.otero@udc.es (P.O.); 2 Department of Evolutionary and Educational Psychology, University of Santiago de Compostela, 15782 Santiago de Compostela, Spain; vanessa.blanco@usc.es; 3 Department of Clinical Psychology and Psychobiology, University of Santiago de Compostela, 15782 Santiago de Compostela, Spain; fernandolino.vazquez@usc.es

**Keywords:** sleep quality, psychological distress, caregiver burden, family caregivers

## Abstract

This study aimed to determine the prevalence and associated factors of poor sleep quality in non-professional caregivers. With this purpose, cross-sectional data were collected from 201 dependent people’s family caregivers using the Pittsburgh Sleep Quality Index (PSQI), the Caregiver Burden Inventory (CBI), the General Health Questionnaire (GHQ-12), and an ad hoc questionnaire to obtain sociodemographic data. A total of 153 family caregivers were categorized as poor sleepers (PSQI > 5), resulting in a prevalence of poor sleep quality of 76.1% (95% CI = 70.5–82.5). Poor sleepers were more likely to care for persons with mental disorders (*χ*^2^ = 7.31; *p* < 0.01) and scored significantly higher on perceived burden (*z* = −4.44; *p* < 0.001), psychological distress (*z* = −6.24; *p* < 0.001), and in all the PSQI subscales (*p* < 0.001), compared with good sleepers (PSQI ≤ 5). By contrast, no differences were found between poor and good sleepers in age, gender, years providing care, and daily hours of care. Multiple linear regression analysis showed that the factors of caregiver burden (*β* = 0.15; *p* < 0.05) and psychological distress (*β* = 0.53; *p* < 0.001) were significantly associated with sleep quality in dependent people’s family caregivers. Cognitive-behavioral strategies to improve sleep quality in the primary health care of family caregivers are suggested.

## 1. Introduction

At the end of 2020, there were more than 1.3 million people in Spain in a situation of officially recognized dependency [1]. Most of these dependent people live at home under the care of a family member and, due to their high degree of dependency, a significant percentage need constant support to perform multiple tasks that span broad domains of activity. For this reason, informal caregiving has become an important public health matter.

In their everyday life, the dependent people’s family caregivers take on a wide range of tasks and responsibilities, ranging from support to carry out essential daily life basic activities, self-care tasks, and household labor, to the provision of emotional and social support, assistance in mobility, and ensuring adherence of the care recipient to their treatment and/or rehabilitation program, among other important direct and indirect care tasks [2]. Consequently, this informal or non-professional care of the family member deprived of autonomy and independence is a very demanding and stressful task that usually produces negative consequences derived from the burden implied by the role of the caregiver, affecting their work, family life, partner relationship, leisure and free time, health, etc.; in short, their life in general. Caregiver burden is the product of the dynamic interaction among care needs of the dependent people, the care situation, and the caregiver’s resources and vulnerabilities [3].

The negative effects of caregiving on the health and physical, psychological, and social well-being of the dependent people’s family caregivers have been clearly demonstrated in the extensive research activity carried out over the last decade [4,5,6,7,8]. Moreover, the amplitude of these negative effects is particularly marked in family caregivers providing high-intensity care or who experience high levels of perceived burden [9]. Among the several health problems shown by the family caregivers, sleep disturbances and difficulties are particularly important and very prevalent [10]. Compared with age- and gender-matched non-caregiver controls, dependent people’s family caregivers show sleep quality impairment, decreased total sleep time, fragmented sleep by frequent nocturnal arousals, greater daytime sleepiness and fatigue, and insomnia symptoms [11,12,13].

Poor sleep quality significantly affects psychological well-being and health-related quality of life. It is a significant cause of deterioration in the caregiver’s health, a risk factor for numerous mental and physical health problems, and, consequently, also negatively affects the caregiving task itself. Nevertheless, despite its importance, sleep dysfunctions in family caregivers are a relatively underrecognized and under-treated problem [14]. In the domain of sleep research in dependent people’s family caregivers, few studies have been conducted to determine the main variables related to sleep quality and, for this reason, the prevalence and associated factors of poor sleep quality are not well understood.

In a previous study carried out by our research team, the existence of a direct relationship between caregiver burden and sleep quality was evidenced [3]. This association was shown in a significant form in family caregivers with high levels of perceived burden compared with both non-caregiver controls (gender and age matched) and family caregivers with low and medium levels of perceived burden. In addition to the perceived burden, other variables may have an important weight to understand the negative effects of care on health and, more particularly, on sleep. In fact, as result of the prolonged and intense care of the dependent people, it can be very difficult to maintain an adequate emotional balance, which is why dependent people’s family caregivers have high levels of psychological distress and a significantly increased risk of psychopathology [15,16].

One way of obtaining information regarding the influence of psychological distress and perceived burden on the sleep quality of caregivers that has not been investigated is to compare the levels of these variables between good sleepers and poor sleepers who are family caregivers. This is the strategy utilized in this cross-sectional study, whose objective was to estimate the prevalence of poor sleep quality in dependent people’s family caregivers and to examine the possible association between poor sleep quality and psychological distress, caregiver burden, and certain sociodemographic characteristics.

## 2. Materials and Methods

### 2.1. Subjects

The study participants were recruited by simple random sampling from the official register of caregivers of the Dependency Service and Personal Autonomy of the Ministry of Social Policy of the Autonomous Community of Galicia, Spain. Subjects were included in the study on the basis of fulfilling the following inclusion criteria: (a) to be a family caregiver (not a professional who charges to provide these services) of a person with officially recognized dependence, and (b) to live at home with the person cared for. The exclusion criteria were: (a) the presence of any difficulty or condition in the communication that could interfere with the usual psychological assessment process, and/or (b) having received psychological or psychiatric treatment in the last two months. Of the 210 family caregivers initially contacted who were deemed eligible for the study, 9 refused to participate due to being too busy (response rate of 95.7%), resulting in a final total sample of 201 dependent people’s family caregivers (87.1% women) with a mean age of 56.15 years (±10.06). Participation in this study was totally voluntary, and all subjects gave written informed consent. The study protocol was reviewed and approved by the Bioethics Committee of the University of Santiago de Compostela (Code number 07092016, Santiago de Compostela, Spain) and conducted according to the Declaration of Helsinki.

### 2.2. Measures

An ad hoc structured questionnaire was utilized to record the following sociodemographic variables of the dependent people’s family caregivers: age, gender, cause of the care receiver dependence (physical disability, mental disorder), years providing care, and daily hours of care.

The Pittsburgh Sleep Quality Index (PSQI) [17], Spanish version [18], was utilized to assess sleep quality. This version has good psychometric properties, displaying an acceptable internal consistency, reliability, validity, and predictive value, being an appropriate assessment tool for epidemiological and clinical research [19]. The PSQI contains 19 items, yielding 7 subscales or components: subjective sleep quality, sleep latency, sleep duration, habitual sleep efficiency, sleep disturbances, use of sleep medications, and daytime dysfunction. Each subscale is scored between 0 and 3; their sum builds the global PSQI score, ranging from 0 to 21, with higher scores reflecting poor sleep quality. Using a cutoff score above 5, the instrument has a diagnostic sensitivity of 89.6% and specificity of 86.5% in distinguishing “good” versus “poor” sleepers [17].

The Spanish version of the Caregiver Burden Inventory (CBI) [20], carried out by Vázquez et al. [21], was employed to assess the caregiver burden. This instrument of 24 items provides a measure of the impact of the burden on various domains of the caregiver’s life. Each item is quantified using a 5-point Likert scale ranging from 0 to 4, so the maximum total score of CBI is 96. Higher scores in the questionnaire reflect greater levels of perceived burden (scores above 24 indicate a need to seek some form of relay or support in the care task, whereas scores above 36 indicate a risk of “burnout”). This Spanish version has an adequate internal consistency (Cronbach’s *α* = 0.89).

Psychological distress was assessed with the 12-item General Health Questionnaire (GHQ-12) [22]. This quick and unobtrusive questionnaire is one of the most widely used screening instruments for psychiatric morbidity and to obtain a general measure of psychological well-being and mental health. Using a GHQ-type score (the most appropriate when the questionnaire is used as a one-dimensional screening tool), the total score ranged from 0 to 12, with higher scores corresponding to greater emotional discomfort. The GHQ-12 has shown adequate reliability and validity for use in the Spanish population [23]. The optimal cut-off point to detect the possible presence of psychopathology is located at 2/3, with a sensitivity of 76% and a specificity of 80% [24].

### 2.3. Procedure

A research protocol was developed to standardize the assessment procedure, detailing the study objectives, design and setting, participants (target population, accessible population, inclusion/exclusion criteria, sampling, recruitment), measures (predictor and outcome variables), bias (non-response, recall bias, selection bias), data analysis strategy, quality control, data management, schedule, and ethical issues. In addition, before starting the study, we conducted a pilot test to refine the measuring instruments and improve the assessment procedure.

Subsequently, dependent people’s family caregivers were contacted through postal mail and phone, explaining the main objectives of the study, and inviting them to participate voluntarily, without receiving any financial compensation. To encourage study participation and minimize loss of subjects over time, the study presentation was done in an engaging and simple manner, guaranteeing the confidentiality of the data obtained, showing empathy with the family caregivers, and treating them with delicacy and respect.

Once the informed consent was signed, three properly trained and experienced clinical psychologists conducted the assessment of the family caregivers, resolving all possible doubts that might arise. More concretely, they collected information on the sociodemographic characteristics previously described and applied the questionnaires (PSQI, CBI, and GHQ-12). The assessment process was completed in approximately 40 min, being carried out face-to-face and in centers close to the home of the participants.

### 2.4. Data Analysis

Statistical analyses were performed using IBM SPSS Statistics 24 (IBM Corp., Armonk, NY, USA). The total study sample was divided into two groups according to the cutoff of global PSQI scores: good sleepers (global PSQI ≤ 5) or poor sleepers (global PSQI > 5). In the descriptive analysis, data are displayed as frequencies and percentages (%) for the categorical variables and as means and standard deviations (SDs) for the continuous variables. The differences between groups were compared using the chi-square test (*χ*^2^) and Mann–Whitney U test for the categorical and continuous variables, respectively. For the estimation of the prevalence of poor sleep quality in family caregivers, a 95% confidence interval (95% CI) was calculated. Finally, with the purpose of identifying the main associated factors with poor sleep quality in family caregivers, a multiple linear regression analysis (enter method) was conducted, using sleep quality (global PSQI score) as the dependent variable. The significance level was set previously at a *p*-value < 0.05 for all comparisons.

## 3. Results

The sociodemographic and clinical characteristics of the total study sample and for the “good sleepers” and “poor sleepers” subgroups are reported in Table 1.

Regarding the whole sample (*n* = 201), the mean age was 56.15 (±10.06) years, with a higher proportion of females (87.1%). These family caregivers had been a mean of 14.48 (±11.68) years providing care to dependent persons as a result of both physical disabilities (58.2%) and mental disorders (41.8%), dedicating a mean of 16.25 (±5.30) daily hours to this task. Finally, it should be noted that the levels of perceived burden were high (mean CBI score = 40.11), similar to the psychological distress (mean GHQ-12 score = 4.11).

A total of 153 family caregivers were defined as poor sleepers, resulting in a prevalence of poor sleep quality of 76.1% (95% CI = 70.5–82.5). Poor sleepers were more likely to care for persons with mental disorders (*p* < 0.01) and scored significantly higher on perceived burden (*p* < 0.001) and psychological distress (*p* < 0.001) compared to the good sleepers group. Conversely, there were no statistically significant differences between the good and poor sleepers groups in terms of age (*p* = 0.36), gender (*p* = 0.58), years providing care (*p* = 0.72), and daily hours of care (*p* = 0.08).

Mean scores (standard deviations) for global and subscales PSQI in the total study sample of “good sleepers” and “poor sleepers” are summarized in Table 2. As seen, pathological difficulties in sleep were appreciated in the total study sample, as reflected by the high score on the global PSQI (8.97 ± 4.27). In total, 23.9% of the study participants were classified as good sleepers, and 76.1% as poor sleepers. The global PSQI score of the poor sleepers (10.60 ± 3.49) was practically 3 times higher than that of good sleepers (3.64 ± 1.05).

Statistically significant differences were observed among good and poor sleepers in all the PSQI subscales (*p* < 0.001), being more pronounced in sleep duration (*z* = −8.22), subjective sleep quality (*z* = −7.64), sleep latency (*z* = −7.51), and habitual sleep efficiency (*z* = −7.41). In fact, the mean total sleep time of the poor sleepers group was lower than 6 daily hours (5.87 ± 1.12), while in the good sleepers group, it was higher than 7 (7.48 ± 0.80). Likewise, the mean sleep onset latency of the poor sleepers group was notably higher than 30 min (45.33 ± 34.46 min), while in the good sleepers group, it was 16.36 min (±11.52).

Finally, a multiple linear regression analysis (enter method) was conducted using sleep quality (global PSQI score) as the dependent variable and all sociodemographic and clinical characteristics (age, gender, cause of the care receiver dependence, years providing care, daily hours of care, caregiver burden, and psychological distress) as independent variables. This analysis revealed that together, the independent variables explained a significant amount of the variance in the extent of sleep quality (*F*_7,193_ = 21.07; *p* < 0.001; *R*^2^ = 0.43; *R*^2^*_Adjusted_*= 0.41). Nevertheless, the CBI score (*β* = 0.15; *p* < 0.05) and the GHQ-12 score (*β* = 0.53; *p* < 0.001) were the only significantly associated variables with sleep quality in dependent people’s family caregivers.

## 4. Discussion

The purpose of this study was to determine the prevalence and associated factors of poor sleep quality in dependent people’s family caregivers. The results obtained show a high prevalence of poor sleep quality. More than 75% of family caregivers were poor sleepers, and poor sleep quality was associated with both higher perceived burden and higher psychological distress. Moreover, the poor sleepers group showed a very high mean score in perceived burden (CBI score = 42.70), which is indicative of a high risk of “burnout”. Moreover, this family caregivers group evidenced greater psychological distress than that shown by the good sleepers group. Specifically, a 4.86 mean score in GHQ-12, well above the cut score, was obtained. This finding indicates the existence of high psychopathological morbidity in this collective, which should be considered in the field of primary health care.

In addition, poor sleepers family caregivers cared for a higher percentage of people with mental disorders and a lower percentage of people with physical disabilities than good sleepers family caregivers. Usually, caring for dependent people with a mental disorder (particularly with dementia) is more difficult, complex, and demanding than caring for dependent people with a physical disability; thus, their care entails a greater burden and psychological distress, which can negatively affect the family caregiver’s health [25]. The concerns related to the dependency of the person cared for, and the care that the person requires at night (for example, due to the presence of frequent disruptive nocturnal behavior), significantly affect the sleep quality of the caregiver and can directly contribute to disturbed sleep. In turn, poor sleep quality influences daily functioning, probably increasing the perceived burden and the psychological distress levels. In this sense, further research studies should be carried out to clarify this bidirectional relationship between sleep and caregiving.

These findings are consistent with previous studies carried out in this research field that have noted a significant relationship between caregiver burden, psychological distress, and poor sleep quality [3,26,27,28,29].

The results of this study reveal that poor sleepers family caregivers not only need to seek some form of relay or support in the care task, but also, and most particularly, require a direct psychological intervention that enables the acquisition of appropriate strategies to more effectively cope with the demands of caregiving. With this purpose, cognitive-behavioral treatment would be a useful and effective intervention to reduce psychopathological morbidity and alleviate the perceived burden that can precipitate, exacerbate, and chronify family caregivers’ sleep problems.

Sleep is essential for the maintenance of human health and well-being. Healthy sleep demands sufficient duration, good quality, and adequate regularity, and the absence of sleep dysfunctions. The poor sleepers family caregivers group, in comparison with the good sleepers group, was characterized by a lower subjective sleep quality, higher sleep latency, lower sleep duration, lower habitual sleep efficiency, higher sleep disturbances, higher use of sleep medications, and higher daytime dysfunction.

Following the recommendations of the consensus statement about the amount of sleep needed to promote health developed by the American Academy of Sleep Medicine (AASM) and the Sleep Research Society (SRS) [30], adults should sleep at least 7 h per night on a regular basis. A total sleep time lower than 7 h is associated with multiple and varied adverse health outcomes. Nevertheless, the mean total sleep time of the poor sleepers family caregivers group was lower than 6 daily hours and their mean sleep onset latency was notably higher than 30 min. For this reason, it is very important to sensitize and educate family caregivers and their healthcare providers on the importance of appropriate sleep duration and quality for health maintenance.

This study was subjected to several limitations. The sleep quality measure was based only on self-report questionnaires without making use of objective assessment procedures, such as polysomnography. Moreover, caregiving is an eminently dynamic process, and the cross-sectional design of the study did not allow an investigation of the changes that are experienced at different points of time, and seriously limited our ability to identify causal relationships among the analyzed variables. Finally, the value of this study would have been greater if possible protective factors had also been studied, determining which strategies can help caregivers cope with the demands of the care situation and maintain adequate and restful sleep. Despite these limitations, this study included reliable, valid, and extensively used clinical assessment procedures, and the findings obtained can help health primary care professionals to identify dependent people’s family caregivers with a higher risk of poor sleep quality. Cognitive-behavioral treatment tailored to these family caregivers may decrease the perceived burden and the psychological distress, improving the sleep and health-related quality of life of this important collective in an effective and efficient manner.

## 5. Conclusions

The results of this study suggest that poor sleep quality is very common in dependent people’s family caregivers and that poor sleepers have higher levels of perceived burden and psychological distress. The development of specific cognitive-behavioral intervention programs in primary care focused on improving sleep quality, optimizing caregiver roles’ performance, and reducing psychological distress should be a priority of future research in this field.

## Figures and Tables

**Table 1:** Sociodemographic and clinical characteristics of the total study sample of “good sleepers” and “poor sleepers”.

	All Subjects (*n* = 201)	Good Sleepers PSQI ≤ 5 (*n* = 48) (23.9%)	Poor Sleepers PSQI > 5 (*n* = 153) (76.1%)	Comparison *χ^2^*_(1)_/*z p*	
Age (years)	56.15 (10.06)	55.15 (9.30)	56.46 (10.32)	−0.92	0.36
Gender					
- Female	175 (87.1%)	43 (89.6%)	132 (86.3%)		
- Male	26 (12.9%)	5 (10.4%)	21 (13.7%)	0.30	0.58
Cause of the care receiver dependence					
- Physical disability	117 (58.2%)	36 (75%)	81 (52.9%)		
- Mental disorder	84 (41.8%)	12 (25%)	72 (47.1%)	7.31	<0.01
Years providing care	14.48 (11.68)	12.83 (8.35)	14.99 (12.54)	−0.36	0.72
Daily hours of care	16.25 (5.30)	15.09 (5.04)	16.55 (5.33)	−1.74	0.08
Perceived burden (CBI)	40.11 (14.99)	31.73 (11.85)	42.70 (14.98)	−4.44	<0.001
Psychological distress (GHQ-12)	4.11 (3.17)	1.66 (2.03)	4.86 (3.10)	−6.24	<0.001

Note: data are expressed as frequency (*n*) and percentage (*%*) for categorical variables and as mean ($\bar{x}$) and standard deviation (SD) for continuous variables. PSQI: Pittsburgh Sleep Quality Index; CBI: Caregiver Burden Inventory; GHQ-12: 12-item General Health Questionnaire.

**Table 2:** Mean scores (standard deviations) for global and subscales PSQI in the total study sample of “good sleepers” and “poor sleepers”.

	All Subjects (*n* = 201)	Good Sleepers PSQI ≤ 5 (*n* = 48) (23.9%)	Poor Sleepers PSQI > 5 (*n* = 153) (76.1%)	Comparison *z p*	
Global PSQI	8.97 (4.27)	3.64 (1.05)	10.60 (3.49)	−10.38	<0.001
Subjective sleep quality	1.42 (0.79)	0.66 (0.48)	1.65 (0.72)	−7.64	<0.001
Sleep latency	1.66 (1.14)	0.55 (0.62)	2.00 (1.05)	−7.51	<0.001
Sleep duration	1.33 (0.91)	0.38 (0.53)	1.62 (0.79)	−8.22	<0.001
Habitual sleep efficiency	1.22 (1.16)	0.17 (0.38)	1.54 (1.12)	−7.41	<0.001
Sleep disturbances	1.43 (0.62)	1.02 (0.44)	1.55 (0.62)	−5.21	<0.001
Use of sleep medications	0.65 (1.14)	0.17 (0.67)	0.80 (1.22)	−3.60	<0.001
Daytime dysfunction	1.29 (0.86)	0.70 (0.62)	1.46 ((0.84)	−5.44	<0.001

Note: PSQI: Pittsburgh Sleep Quality Index.

## Data Availability

The data presented in this study are available on request from the corresponding author. The data are not publicly available due to confidentiality issues.
